# 3-Oxo-2,3-dihydro-1*H*-inden-4-yl acetate

**DOI:** 10.1107/S1600536812041293

**Published:** 2012-10-06

**Authors:** Hong-Yi Lin, Che-Wei Chang, Hsing-Yang Tsai, Ming-Hui Luo, Kew-Yu Chen

**Affiliations:** a Department of Chemical Engineering, Feng Chia University, 40724 Taichung, Taiwan

## Abstract

In the title compound, C_11_H_10_O_3_, the 1-indanone unit is essentially planar (r.m.s. deviation = 0.036 Å). In the crystal, mol­ecules are linked by non-classical C—H⋯O hydrogen bonds, forming a *C*(6) chain along [010].

## Related literature

For the preparation of the title compound, see: Rahimizadeh *et al.* (2010 ▶). For applications of indanone derivatives, see: Borbone *et al.* (2011 ▶); Borge *et al.* (2010 ▶); Cai *et al.* (2005 ▶); Cui *et al.* (2009 ▶); Fu & Wang (2008 ▶); Li *et al.* (2009 ▶); Sousa *et al.* (2011 ▶); Tang *et al.* (2011 ▶); Yu *et al.* (2011 ▶). For related structures, see: Ali *et al.* (2010*a* ▶,*b* ▶,*c* ▶,*d* ▶); Chen *et al.* (2011*a* ▶,*b* ▶). For C—H⋯O hydrogen bonds, see: Li *et al.* (2011*a* ▶,*b* ▶); Wang & Chen (2011 ▶); Xi *et al.* (2010 ▶). For graph-set theory, see: Bernstein *et al.* (1995 ▶). 
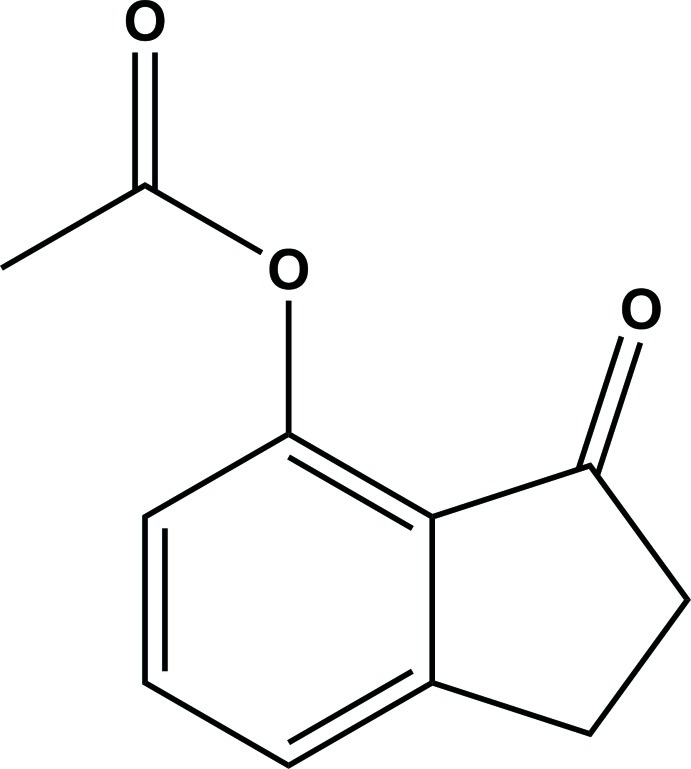


## Experimental

### 

#### Crystal data

C_11_H_10_O_3_
*M* *_r_* = 190.19
Orthorhombic, 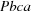

*a* = 9.8514 (10) Å
*b* = 8.9757 (7) Å
*c* = 21.917 (3) Å
*V* = 1938.0 (3) Å^3^
*Z* = 8
Mo *K*α radiation
μ = 0.10 mm^−1^
*T* = 297 K
0.76 × 0.60 × 0.28 mm

#### Data collection

Bruker SMART CCD area-detector diffractometer
Absorption correction: multi-scan (*SADABS*; Bruker, 2001 ▶) *T*_min_ = 0.761, *T*_max_ = 1.000
8705 measured reflections
2339 independent reflections
1302 reflections with *I* > 2σ(*I*)
*R*_int_ = 0.036

#### Refinement

*R*[*F* ^2^ > 2σ(*F* ^2^)] = 0.087
*wR*(*F* ^2^) = 0.231
*S* = 1.10
2339 reflections
127 parameters
H-atom parameters constrained
Δρ_max_ = 0.23 e Å^−3^
Δρ_min_ = −0.21 e Å^−3^

### 

Data collection: *SMART* (Bruker, 2001 ▶); cell refinement: *SAINT* (Bruker, 2001 ▶); data reduction: *SAINT*; program(s) used to solve structure: *SHELXS97* (Sheldrick, 2008 ▶); program(s) used to refine structure: *SHELXL97* (Sheldrick, 2008 ▶); molecular graphics: *ORTEP-3 for Windows* (Farrugia, 1997 ▶); software used to prepare material for publication: *WinGX* publication routines (Farrugia, 1999 ▶).

## Supplementary Material

Crystal structure: contains datablock(s) I. DOI: 10.1107/S1600536812041293/ff2083sup1.cif

Structure factors: contains datablock(s) I. DOI: 10.1107/S1600536812041293/ff2083Isup2.hkl

Supplementary material file. DOI: 10.1107/S1600536812041293/ff2083Isup3.cml

Additional supplementary materials:  crystallographic information; 3D view; checkCIF report

## Figures and Tables

**Table 1:** Hydrogen-bond geometry (Å, °)

*D*—H⋯*A*	*D*—H	H⋯*A*	*D*⋯*A*	*D*—H⋯*A*
C8—H8*A*⋯O3^i^	0.93	2.46	3.223 (5)	139

Symmetry code: (i) 
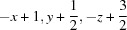
.

**Fig. 1.**
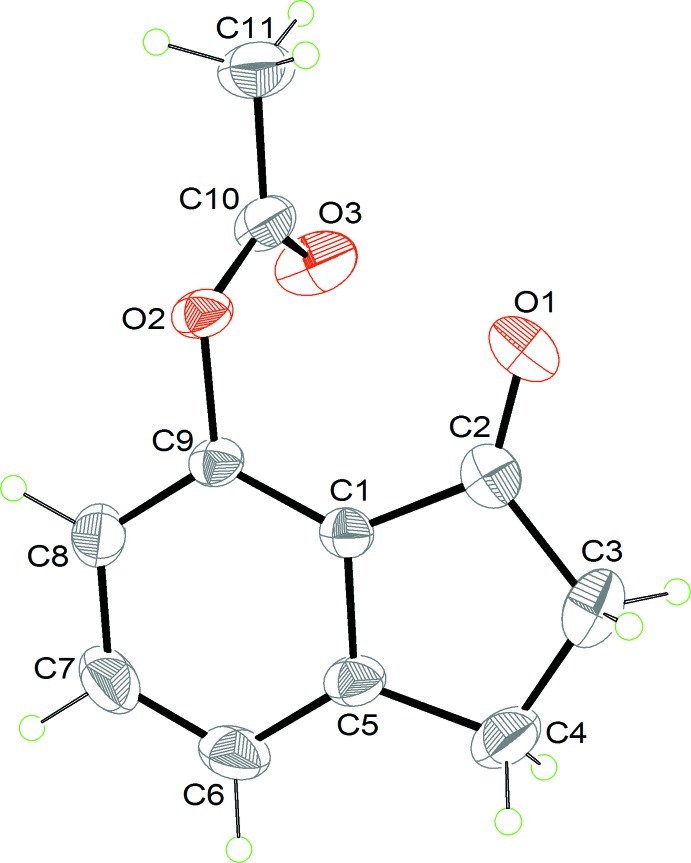
The molecular structure of the title compound, showing 50% probability displacement ellipsoids.

**Fig. 2.**
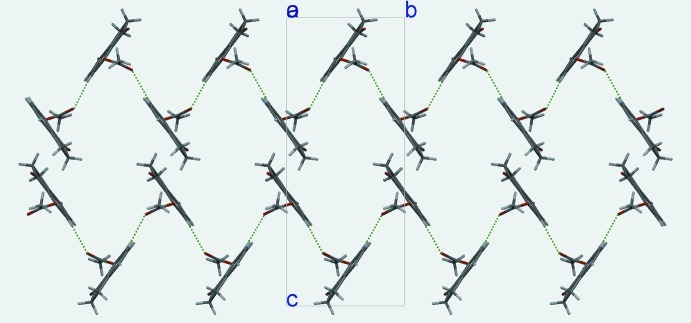
A section of the crystal packing of the title compound, viewed down the a axis. Green dashed lines denote the intermolecular C8—H8A···O3 hydrogen bonds.

## supplementary crystallographic information

### Comment

Indanone derivatives are some of the most widely used organic compounds (Tang *et al.*, 2011). They are used as pigments and dyes (Cui *et al.*, 2009; Li *et al.*, 2009), intermediates in organic synthesis (Borbone *et al.*, 2011; Borge *et al.*, 2010; Fu & Wang, 2008; Yu *et al.*, 2011) and exhibit a wide variety of biological activities (Sousa *et al.*, 2011). Furthermore, 1-indanones were important precursors in the regiospecific synthesis of 2-fluoro-1-naphthols (Cai *et al.*, 2005).

The molecular structure of the title compound is shown in Figure 1. The 1-indaneone moiety is essentially planar (r.m.s. deviation = 0.036 Å), which is consistent with previous studies (Ali *et al.*, 2010*a*,*b*,*c*,*d*; Chen *et al.*, 2011*a*,*b*). In the crystal (Fig. 2), molecules are linked by nonclassical intermolecular C—H···O (Li *et al.*, 2011*a*,*b*; Wang *et al.*, 2011; Xi *et al.*, 2010) hydrogen bonds (Table 1) to form an infinite one-dimensional chain along [010], generating a C(6) motif (Bernstein *et al.*, 1995).

### Experimental

The title compound was synthesized by the acetylation of 7-hydroxyindan-1-one with acetyl chloride (Rahimizadeh *et al.*, 2010). Colorless parallelepiped-shaped crystals suitable for the crystallographic studies reported here were isolated over a period of five weeks by slow evaporation from a chloroform solution.

### Refinement

The C bound H atoms were positioned geometrically (C—H = 0.93–0.97 Å) and allowed to ride on their parent atoms, with *U*_iso_(H) = 1.2–1.5*U*_eq_(C).

### Crystal data

**Table:**

C_11_H_10_O_3_	*F*(000) = 800
*M**_r_* = 190.19	*D*_x_ = 1.304 Mg m^−^^3^
Orthorhombic, *P**b**c**a*	Mo *K*α radiation, λ = 0.71073 Å
Hall symbol: -P 2ac 2ab	Cell parameters from 2935 reflections
*a* = 9.8514 (10) Å	θ = 3.1–29.2°
*b* = 8.9757 (7) Å	µ = 0.10 mm^−^^1^
*c* = 21.917 (3) Å	*T* = 297 K
*V* = 1938.0 (3) Å^3^	Parallelepiped, colourless
*Z* = 8	0.76 × 0.60 × 0.28 mm

### Data collection

**Table:**

Bruker SMART CCD area-detector diffractometer	2339 independent reflections
Radiation source: fine-focus sealed tube	1302 reflections with *I* > 2σ(*I*)
Graphite monochromator	*R*_int_ = 0.036
ω scans	θ_max_ = 29.3°, θ_min_ = 3.2°
Absorption correction: multi-scan (*SADABS*; Bruker, 2001)	*h* = −13→13
*T*_min_ = 0.761, *T*_max_ = 1.000	*k* = −12→12
8705 measured reflections	*l* = −30→30

### Refinement

**Table:**

Refinement on *F*^2^	Primary atom site location: structure-invariant direct methods
Least-squares matrix: full	Secondary atom site location: difference Fourier map
*R*[*F*^2^ > 2σ(*F*^2^)] = 0.087	Hydrogen site location: inferred from neighbouring sites
*wR*(*F*^2^) = 0.231	H-atom parameters constrained
*S* = 1.10	*w* = 1/[σ^2^(*F*_o_^2^) + (0.074*P*)^2^ + 2.2948*P*] where *P* = (*F*_o_^2^ + 2*F*_c_^2^)/3
2339 reflections	(Δ/σ)_max_ = 0.001
127 parameters	Δρ_max_ = 0.23 e Å^−^^3^
0 restraints	Δρ_min_ = −0.21 e Å^−^^3^

### Special details

**Table:**

Geometry. All e.s.d.'s (except the e.s.d. in the dihedral angle between two l.s. planes) are estimated using the full covariance matrix. The cell e.s.d.'s are taken into account individually in the estimation of e.s.d.'s in distances, angles and torsion angles; correlations between e.s.d.'s in cell parameters are only used when they are defined by crystal symmetry. An approximate (isotropic) treatment of cell e.s.d.'s is used for estimating e.s.d.'s involving l.s. planes.
Refinement. Refinement of *F*^2^ against ALL reflections. The weighted *R*-factor *wR* and goodness of fit *S* are based on *F*^2^, conventional *R*-factors *R* are based on *F*, with *F* set to zero for negative *F*^2^. The threshold expression of *F*^2^ > σ(*F*^2^) is used only for calculating *R*-factors(gt) *etc*. and is not relevant to the choice of reflections for refinement. *R*-factors based on *F*^2^ are statistically about twice as large as those based on *F*, and *R*- factors based on ALL data will be even larger.

### Fractional atomic coordinates and isotropic or equivalent isotropic displacement parameters (Å^2^)

**Table:**

	*x*	*y*	*z*	*U*_iso_*/*U*_eq_	
O1	0.4675 (3)	−0.1591 (3)	0.54019 (14)	0.0748 (9)	
O2	0.4074 (2)	0.0430 (2)	0.64503 (12)	0.0495 (7)	
O3	0.4063 (3)	−0.1886 (3)	0.68244 (16)	0.0790 (10)	
C1	0.6245 (3)	−0.0223 (3)	0.60176 (15)	0.0398 (8)	
C2	0.5807 (4)	−0.1175 (4)	0.55084 (17)	0.0525 (9)	
C3	0.7066 (5)	−0.1530 (5)	0.5139 (2)	0.0708 (12)	
H3A	0.7156	−0.2598	0.5086	0.085*	
H3B	0.7013	−0.1067	0.4740	0.085*	
C4	0.8266 (4)	−0.0918 (5)	0.5494 (2)	0.0703 (13)	
H4A	0.8833	−0.1720	0.5645	0.084*	
H4B	0.8812	−0.0269	0.5239	0.084*	
C5	0.7649 (3)	−0.0065 (4)	0.60117 (17)	0.0495 (9)	
C6	0.8271 (4)	0.0804 (5)	0.6447 (2)	0.0670 (12)	
H6A	0.9208	0.0928	0.6442	0.080*	
C7	0.7502 (5)	0.1487 (5)	0.6887 (2)	0.0715 (12)	
H7A	0.7929	0.2063	0.7183	0.086*	
C8	0.6107 (4)	0.1338 (4)	0.68999 (18)	0.0566 (10)	
H8A	0.5596	0.1815	0.7198	0.068*	
C9	0.5494 (3)	0.0480 (3)	0.64677 (16)	0.0398 (8)	
C10	0.3463 (4)	−0.0857 (4)	0.66149 (19)	0.0551 (10)	
C11	0.1978 (4)	−0.0793 (5)	0.6504 (3)	0.0788 (14)	
H11A	0.1572	−0.1721	0.6623	0.118*	
H11B	0.1813	−0.0620	0.6078	0.118*	
H11C	0.1590	0.0002	0.6739	0.118*	

### Atomic displacement parameters (Å^2^)

**Table:**

	*U*^11^	*U*^22^	*U*^33^	*U*^12^	*U*^13^	*U*^23^
O1	0.0656 (19)	0.0770 (19)	0.082 (2)	−0.0191 (16)	−0.0143 (16)	−0.0153 (16)
O2	0.0343 (13)	0.0359 (12)	0.0784 (18)	0.0037 (10)	0.0070 (12)	0.0055 (12)
O3	0.0662 (19)	0.0619 (18)	0.109 (3)	0.0096 (15)	0.0190 (18)	0.0358 (17)
C1	0.0376 (17)	0.0323 (15)	0.0494 (19)	0.0003 (13)	−0.0006 (15)	0.0039 (14)
C2	0.059 (2)	0.0463 (19)	0.052 (2)	0.0001 (18)	−0.0060 (19)	0.0029 (17)
C3	0.083 (3)	0.065 (3)	0.065 (3)	0.019 (2)	0.014 (2)	−0.004 (2)
C4	0.055 (2)	0.066 (3)	0.090 (3)	0.011 (2)	0.024 (2)	0.000 (2)
C5	0.0359 (18)	0.0455 (18)	0.067 (2)	0.0046 (15)	0.0031 (17)	0.0047 (17)
C6	0.039 (2)	0.064 (2)	0.098 (3)	−0.0033 (19)	−0.014 (2)	−0.001 (2)
C7	0.065 (3)	0.065 (3)	0.084 (3)	−0.005 (2)	−0.025 (2)	−0.021 (2)
C8	0.057 (2)	0.055 (2)	0.058 (2)	0.0073 (19)	−0.0038 (19)	−0.0119 (19)
C9	0.0329 (17)	0.0351 (15)	0.051 (2)	0.0057 (13)	−0.0009 (14)	0.0054 (15)
C10	0.047 (2)	0.048 (2)	0.071 (3)	0.0038 (17)	0.0154 (19)	0.0047 (19)
C11	0.048 (2)	0.065 (3)	0.123 (4)	−0.005 (2)	0.012 (3)	0.003 (3)

### Geometric parameters (Å, º)

**Table:**

O1—C2	1.200 (4)	C4—H4B	0.9700
O2—C10	1.351 (4)	C5—C6	1.376 (6)
O2—C9	1.399 (4)	C6—C7	1.371 (6)
O3—C10	1.189 (4)	C6—H6A	0.9300
C1—C5	1.391 (5)	C7—C8	1.381 (6)
C1—C9	1.385 (4)	C7—H7A	0.9300
C1—C2	1.470 (5)	C8—C9	1.362 (5)
C2—C3	1.514 (6)	C8—H8A	0.9300
C3—C4	1.518 (6)	C10—C11	1.484 (5)
C3—H3A	0.9700	C11—H11A	0.9600
C3—H3B	0.9700	C11—H11B	0.9600
C4—C5	1.497 (5)	C11—H11C	0.9600
C4—H4A	0.9700		
			
C10—O2—C9	117.7 (3)	C5—C6—C7	119.7 (4)
C5—C1—C9	119.4 (3)	C5—C6—H6A	120.2
C5—C1—C2	110.1 (3)	C7—C6—H6A	120.2
C9—C1—C2	130.5 (3)	C6—C7—C8	121.4 (4)
O1—C2—C1	127.0 (4)	C6—C7—H7A	119.3
O1—C2—C3	126.3 (4)	C8—C7—H7A	119.3
C1—C2—C3	106.7 (3)	C9—C8—C7	118.8 (4)
C4—C3—C2	106.7 (3)	C9—C8—H8A	120.6
C4—C3—H3A	110.4	C7—C8—H8A	120.6
C2—C3—H3A	110.4	C8—C9—C1	121.1 (3)
C4—C3—H3B	110.4	C8—C9—O2	118.7 (3)
C2—C3—H3B	110.4	C1—C9—O2	120.0 (3)
H3A—C3—H3B	108.6	O3—C10—O2	123.1 (3)
C5—C4—C3	104.9 (3)	O3—C10—C11	125.7 (4)
C5—C4—H4A	110.8	O2—C10—C11	111.2 (3)
C3—C4—H4A	110.8	C10—C11—H11A	109.5
C5—C4—H4B	110.8	C10—C11—H11B	109.5
C3—C4—H4B	110.8	H11A—C11—H11B	109.5
H4A—C4—H4B	108.8	C10—C11—H11C	109.5
C6—C5—C1	119.6 (4)	H11A—C11—H11C	109.5
C6—C5—C4	129.4 (4)	H11B—C11—H11C	109.5
C1—C5—C4	111.0 (3)		
			
C5—C1—C2—O1	176.0 (4)	C4—C5—C6—C7	−179.7 (4)
C9—C1—C2—O1	−3.9 (6)	C5—C6—C7—C8	−0.8 (7)
C5—C1—C2—C3	−4.2 (4)	C6—C7—C8—C9	0.6 (7)
C9—C1—C2—C3	175.9 (3)	C7—C8—C9—C1	−0.6 (6)
O1—C2—C3—C4	−173.2 (4)	C7—C8—C9—O2	−174.7 (3)
C1—C2—C3—C4	7.0 (4)	C5—C1—C9—C8	0.8 (5)
C2—C3—C4—C5	−7.0 (4)	C2—C1—C9—C8	−179.3 (3)
C9—C1—C5—C6	−1.0 (5)	C5—C1—C9—O2	174.8 (3)
C2—C1—C5—C6	179.1 (3)	C2—C1—C9—O2	−5.2 (5)
C9—C1—C5—C4	179.6 (3)	C10—O2—C9—C8	−110.5 (4)
C2—C1—C5—C4	−0.4 (4)	C10—O2—C9—C1	75.4 (4)
C3—C4—C5—C6	−174.7 (4)	C9—O2—C10—O3	7.1 (6)
C3—C4—C5—C1	4.7 (5)	C9—O2—C10—C11	−173.3 (3)
C1—C5—C6—C7	1.0 (6)		

### Hydrogen-bond geometry (Å, º)

**Table:**

*D*—H···*A*	*D*—H	H···*A*	*D*···*A*	*D*—H···*A*
C8—H8*A*···O3^i^	0.93	2.46	3.223 (5)	139

Symmetry code: (i) −*x*+1, *y*+1/2, −*z*+3/2.
